# The Sand Club

**Published:** 1874-01

**Authors:** 


The Sand Club.—This is a weapon used by rowdies and criminals in San Francisco, resembling in principle the sand-bag used by the same sort of scoundrels in New York. The sand-club is formed by filling an eel-skin with sand. When this instrument was first brought into use, the authorities were greatly puzzled by deaths, apparently from violence, yet no marks could be found on the outside of the body. A burglar was finally captured with a sand-club in his possession, made out of an eel-skin stuffed with sand. Being closely questioned he explained its use. When the victim is struck, for instance, on the head, he drops insensible, and soon dies from congestion of the brain. Often the skull suffers no injury from the stroke; and if the person struck recovers sensibility, he gradually relapses into a condition of idiocy. Sometimes a man struck in the body will be knocked down by the peculiar force of the blow, and feel no immediate results from it. In a few weeks, however, the flesh will begin to mortify under the line of the blow, and rot down to the bone.—*Scientific American.*

